# Supplementary material for: N6-methyladenosine helps Apostichopus japonicus resist Vibrio splendidus infection by targeting coelomocyte autophagy via the AjULK-AjYTHDF/AjEEF-1α axis
Source: Commun Biol. 2023 May 20;6:547. doi: 10.1038/s42003-023-04929-5 (PMC10199899; doi:10.1038/s42003-023-04929-5)
Supplement: Supplementary file 2 — Supplementary Information [file 42003_2023_4929_MOESM2_ESM.pdf]

Supplementary Table 1 Primers used in this study

Names	Sequences (5'-3')	Application
AjMETTL-F	GAGCTCATGTCGAGCACAGGTGACATCG	Vector construct
AjMETTL3-R	ATTTGCGGCCGCTAACTCGGTCCTAAACTTGTCGGT	
Luc-AjULK-1F	CTCGAGCTAACCCCCTAAGAAAACACACAGA	
Luc-AjULK-1R	GTTTAAACATAAATGGCAGACTGATTGGCTC	
Luc-AjULK-2F	CTCGAGTCTTCAGGGTTTGAGGAAATGTG	
Luc-AjULK-2R	GTTTAAACGTCGCTGGTCTAGTATGTTCTTCAA	
AjYTHDF-F	GAATTCCCATGTCTGGAGCCGGCAATGTTGAAC	
AjYTHDF-R	GCGGCCGCTCAGTTGCGATGACCGCCACGAG	
AjEEF-1α-F	GAATTCATGGGTAAAGAATTGATTCATATCA	
AjEEF-1α-R	GCGGCCGCTCACTTCTTGGCTATTTTGGC	
qAjMETTL3-F	GATGTCATTGTTGCTGAAGTCCG	Real-time PCR
qAjMETTL3-R	AACTTGTCGGTGGTTTCATTGCG	
qAjULK-F	GCCAAGCAGTCTTCAGTTAGGAT	
qAjULK-R	GGCGTCGTCTCTGTGTGTTT	
qYTHDF-F	ATGGAACACTGGAGACAAACCC	
qYTHDF-R	CCGTGATGATTCTGAGAGTTGG	
m6A-qAjULK-F	AGAGGTGGAGAAAGACTCGGTT	
m6A-qAjULK-R	GTGAACCTGCTTCTCTAGGAATATC	
AjULK-MUT-1F	TGGCCCAGCATCGAGTCATGATTCTA	
AjULK-MUT-1R	ACTCGATGCTGGGCCAGCTTGGATGG	
AjULK-MUT-2F	AATGCTGCTACACGGTCTCTGCTTAC	Site mutation
AjULK-MUT-2R	ACCGTGTAGCAGCATTTGGGCTGAAT	
AjYTHDF-MUT-F	CTTTTTATTATCGCGAGTGCCTCGGAAGACGACATCCA<br>CCGTTC	
AjYTHDF-MUT-R	CCGAGGCACTCGCGATAATAAAAAGTCGGGCGTTCTT<br>CAACTCC	
Negative control	UUCUCCGAACGUGUCACGUTT<br>ACGUGACACGUUCGGAGAATT	
AjULK siRNA	GCGGUUCACUUUAUACAUTT<br>AUGUAUAAAGGUGAACCGCTT	RNA interference
AjMETT3 siRNA	CCAGCACAAAUUUCAUCAUTT<br>AUGAUGAAAUUUGUGCUGGTT	
AjYTHDF siRNA	GUCGACUGGUGGUGAUAAUTT<br>AUUAUCACCACCAGUCGACTT	
AjEEF-1α siRNA	CGUGCAAAUUUGAAGAUAUTT<br>AUAUCUUCAAAUUUGCACGTT	
AjULK WT probe	AGGUAUCAUUCAGCCCAAAUGCUGCUACACGGA^{m6A}C<br>UCUGCUUACAGGCTAC-Biotin	RNA pull down and RNA EMSA
AjULK Mut probe	AGGUAUCAUUCAGCCCAAAUGCUGCUACACGGUCUC<br>UGCUUACAGGCTAC-Biotin	

Supplementary Table 2 Antibodies information in this study

Antibodies	Isotype	Use	Product no.	Source
AjMETTL3 antibody	Mouse	WB:(1:500)		Antiserum
AjYTHDF antibody	Mouse	WB:(1:500)<br>IP:(1:200)		Antiserum
AjATG13 antibody	Mouse	WB:(1:500)		Antiserum
AjBeclin-1 antibody	Mouse	WB:(1:500)		Antiserum
Flagellum antibody	Mouse	WB:(1:500)		Antiserum
LAMP	Mouse	WB:(1:2000)	AG2482	Beyotime
AjULK antibody	Rabbit	WB:(1:2000)	T56902S	Abmart
Ajp62 antibody	Rabbit	WB:(1:2000)	P113161	KleanAB
AjLC3 antibody	Rabbit	WB:(1:2000)	EPR18709	Abcam
AjEEF-1α antibody	Rabbit	WB:(1:2000)<br>IP:(1:200)	AF6765	Beyotime
β-actin	Rabbit	WB:(1:2000)	P100003	KleanAB

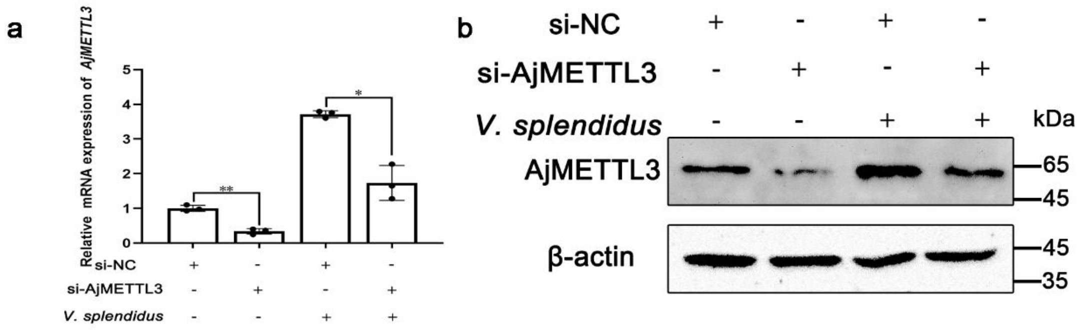

Supplementary Figure 1 siRNA efficiency of AjMETTL3 as determined using qRT-PCR or western blotting. After *A. japonicus* were infected or uninfected by 1 × 10^7 CFU/mL *V. splendidus* for 24 h in AjMETTL3 knockdown condition, qRT-PCR (a) and Western blot (b) analysis of mRNA and protein levels of *AjMETTL3* in coelomocytes, respectively, were conducted. The mRNA and protein levels of AjMETTL3 in the si-AjMETTL3 group were significantly down-regulated compared with those of the proteins in the si-NC group. Under *V. splendidus* infection conditions, a similar phenomenon was observed. The data are presented as means ± SDs (n = 3) relative to the si-NC group and shown in bar graphs. Asterisks indicate significant differences between groups: *$p < 0.05$ and **$p < 0.01$ by one-way ANOVA. The bands presented were from two blot membranes and same quality of protein (50 µg) were loaded into each lane. Error bars represent SDs.

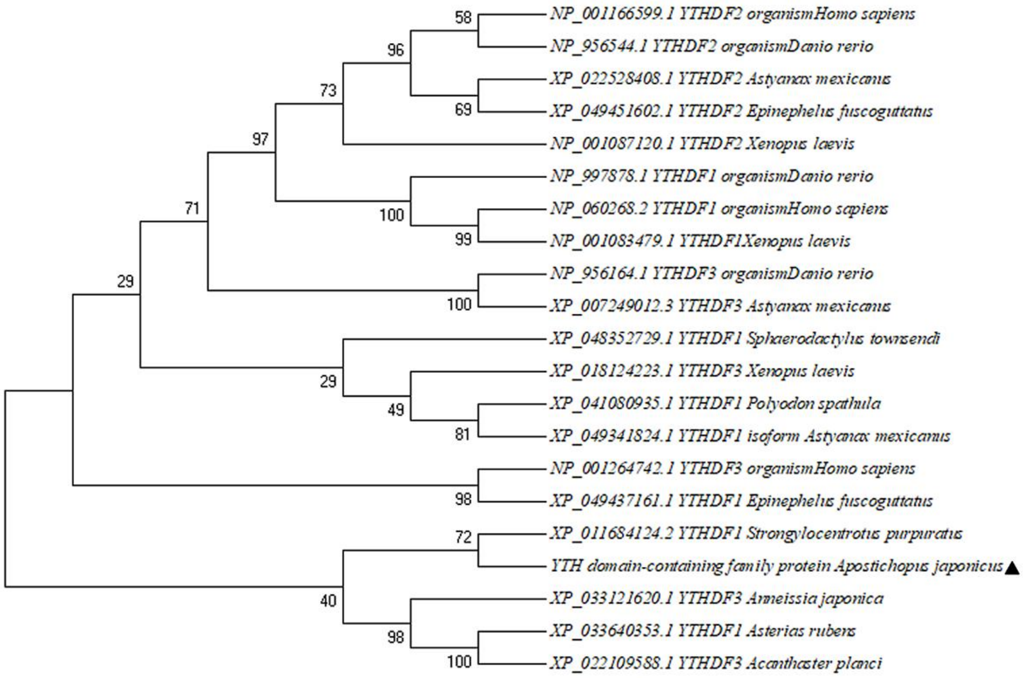

Supplementary Figure 2 Homologous analysis of AjYTHDF. The phylogenic tree constructed using MEGA 7.0 to analyze the evolutionary relationship between *Aj*YTHDF and other YTHDF family members. *NP_060268.2* YTHDF1 [organism=*Homo sapiens*]; *NP_001166599.1* YTHDF2 [organism=*Homo sapiens*]; *NP_001264742.1* YTHDF3 [organism=*Homo sapiens*]; *NP_997878.1* YTHDF1 [organism=*Danio rerio*]; *NP_956544.1* YTHDF2 [organism=*Danio rerio*]; *NP_956164.1* YTHDF3 [organism=*Danio rerio*]; *XP_011684124.2* YTHDF1 [*Strongylocentrotus purpuratus*]; *XP_048352729.1* YTHDF1 [*Sphaerodactylus townsendi*]; *XP_041080935.1* YTHDF1 [*Polyodon spathula*]; *XP_033640353.1* YTHDF1 [*Asterias rubens*]; *XP_022109588.1* YTHDF3 [*Acanthaster planci*]; *XP_033121620.1* YTHDF3 [*Anneissia japonica*]; *XP_022528408.1* YTHDF2 [*Astyanax mexicanus*]; *XP_049341824.1* YTHDF1 isoform [*Astyanax mexicanus*]; *XP_049437161.1* YTHDF1 [*Epinephelus fuscoguttatus*]; *NP_001083479.1* YTHDF1[*Xenopus laevis*]; *NP_001087120.1* YTHDF2 [*Xenopus laevis*]; *XP_007249012.3* YTHDF3 *[Astyanax mexicanus]; XP_018124223.1* YTHDF3 [*Xenopus laevis*]; *XP_049451602.1* YTHDF2 [*Epinephelus fuscoguttatus*].

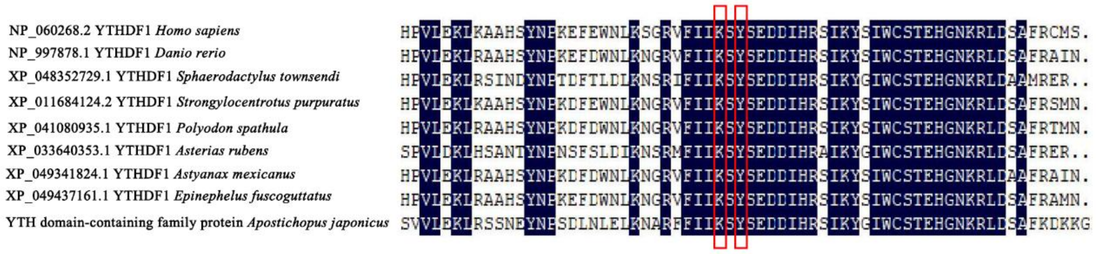

Supplementary Figure 3 Analysis of the key sites for YTH domain bound with m6A-modified mRNA. Multiple sequence alignment found the K464 and Y466 sites in AjYTHDF were conserved to K395 and Y397, respectively.

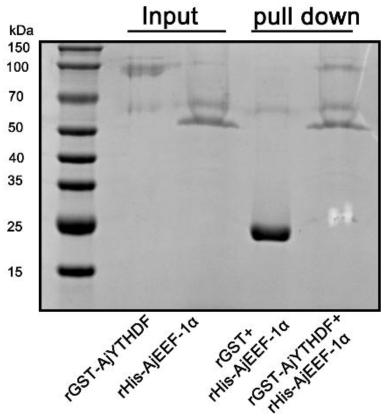

Supplementary Figure 4 Interactions between GST-tagged AjYTHDF and His-tagged AjEEF-1α were detected using pull down assays. GST pull-down assay found AjYTHDF could interact directly with AjEEF-1α.

Full length uncropped original western blots used in the manuscript.

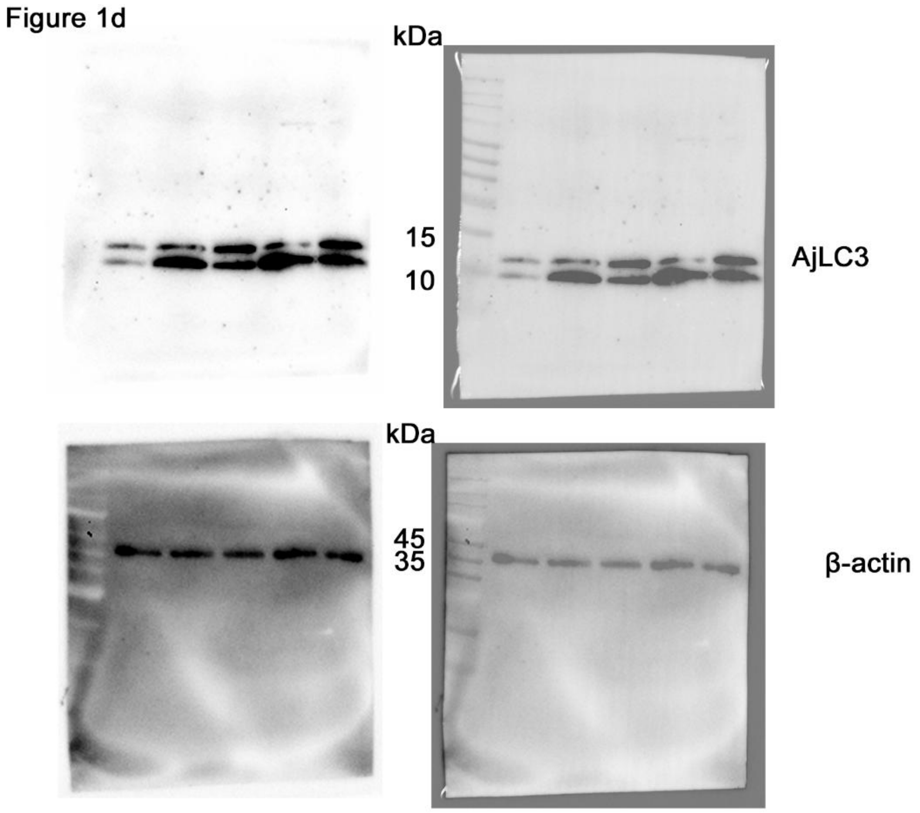

Supplementary Figure 5. Western blots of Figure 1d. The blot image is from same blot membranes.

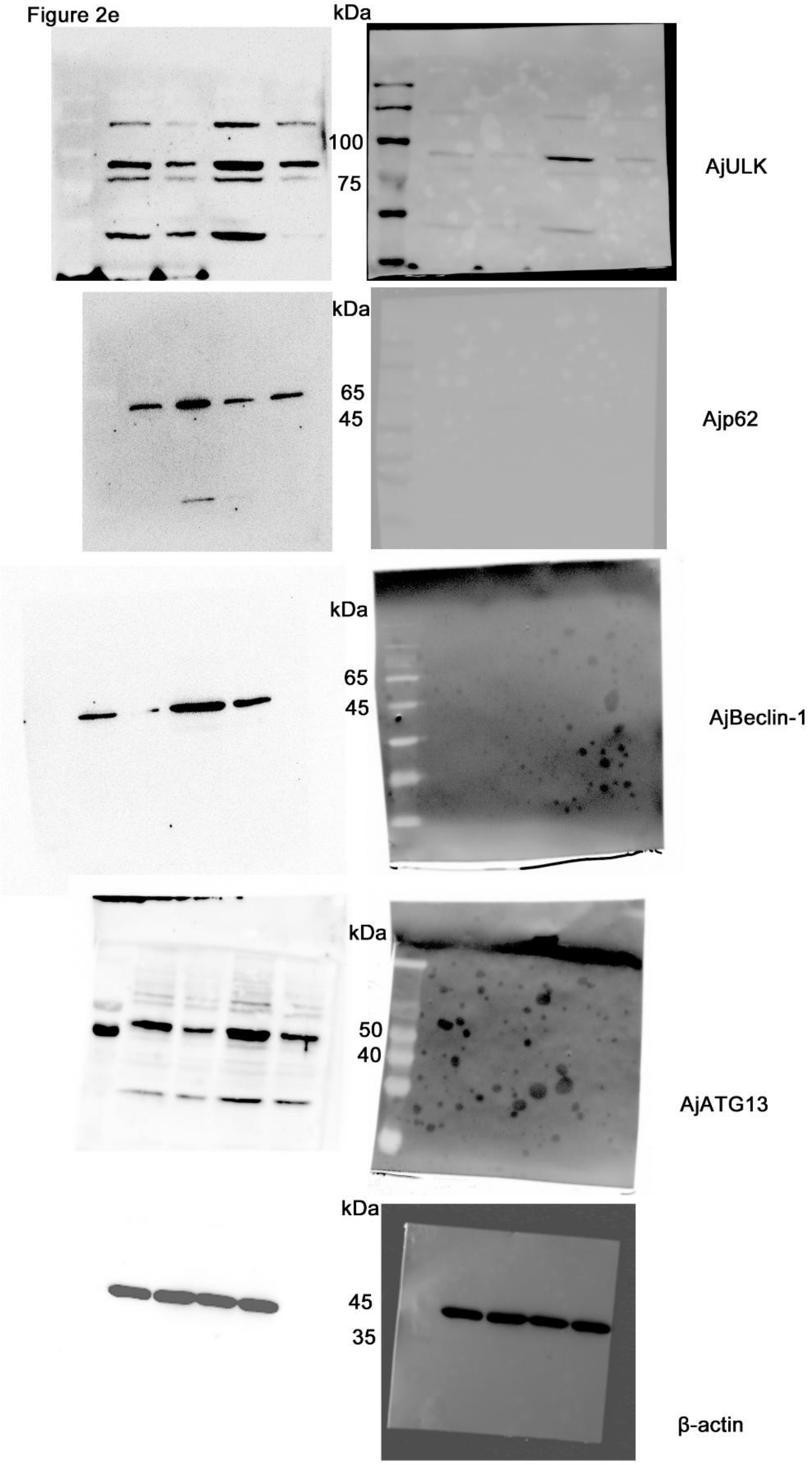

Supplementary Figure 6. Western blots of Figure 2e. The blot image is from five blot membranes.

Figure 3b

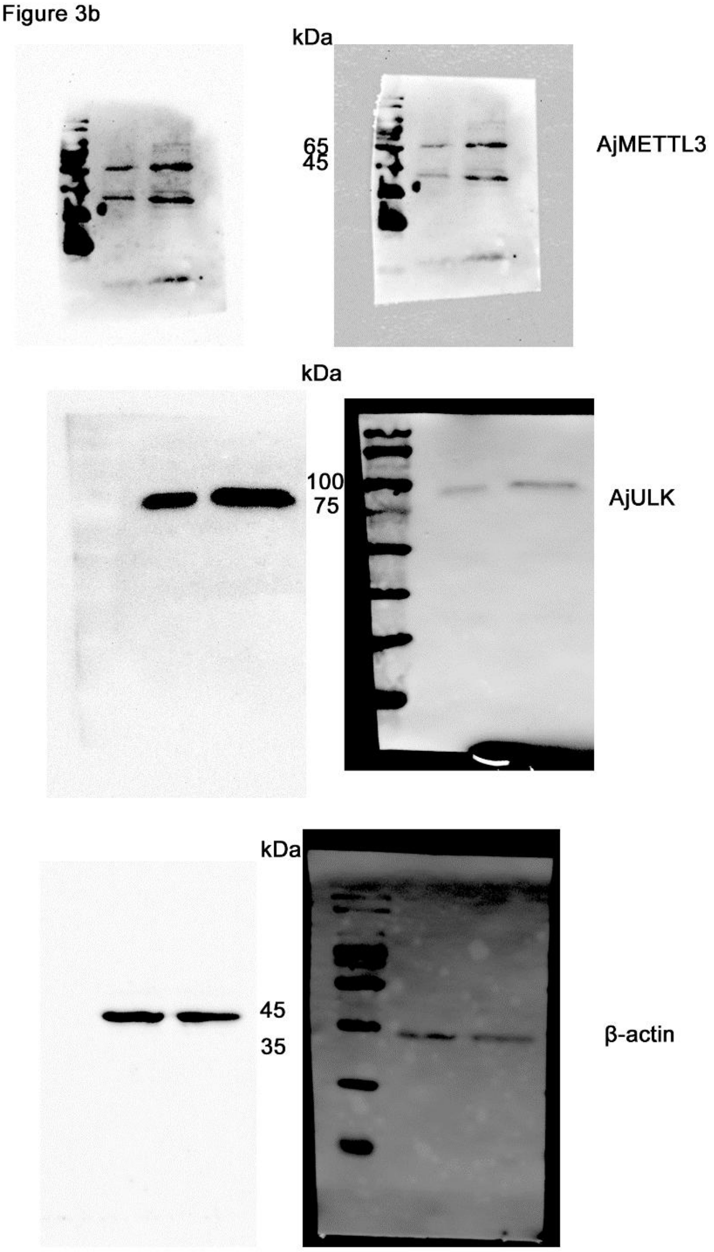

Supplementary Figure 7. Western blots of Figure 3b. The blot image is from three blot membranes.

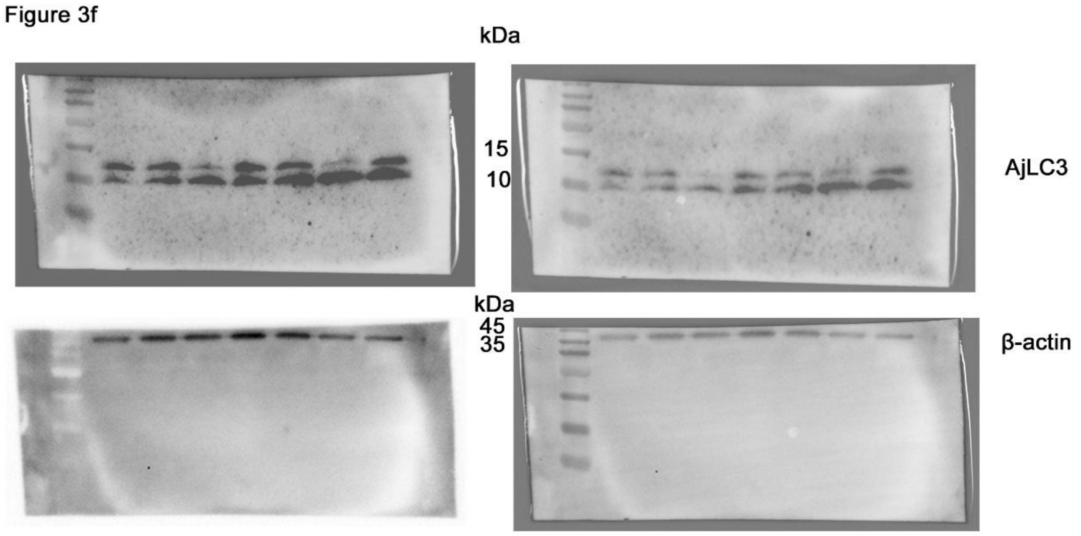

Supplementary Figure 8. Western blots of Figure 3f. The blot image is from same blot membranes.

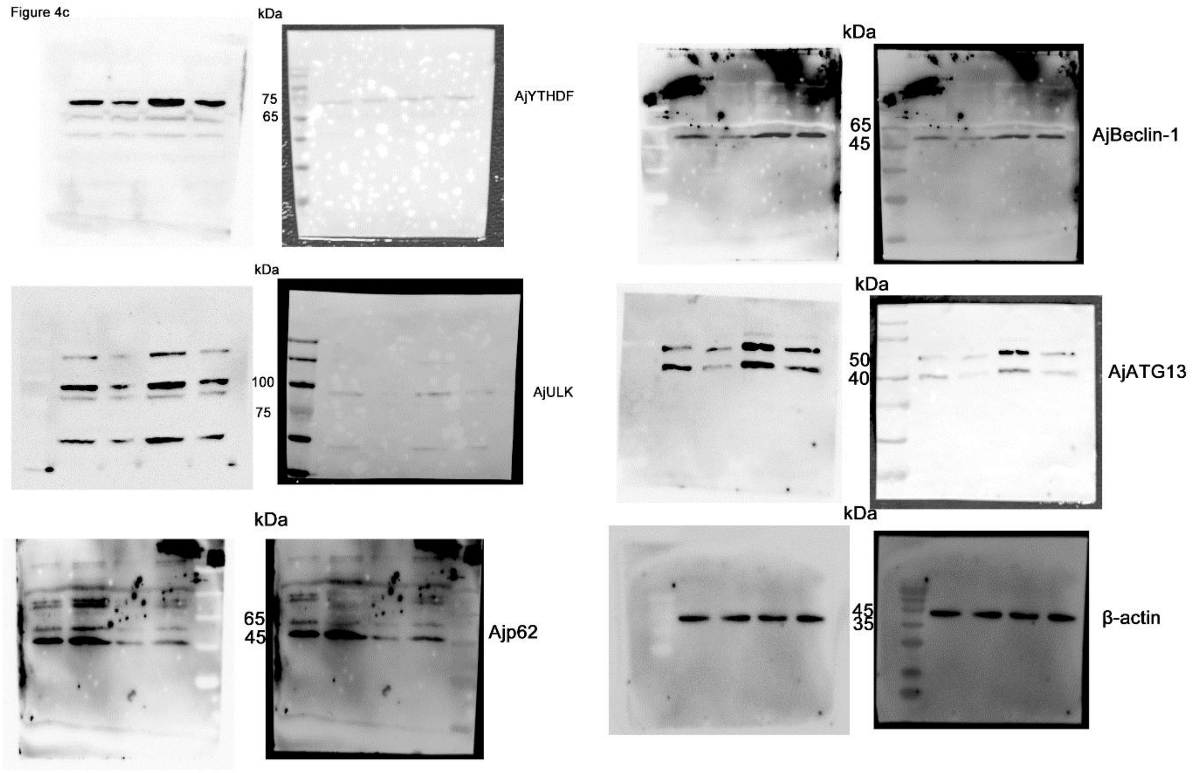

Supplementary Figure 9. Western blots of Figure 4c. The blot image is from six blot membranes.

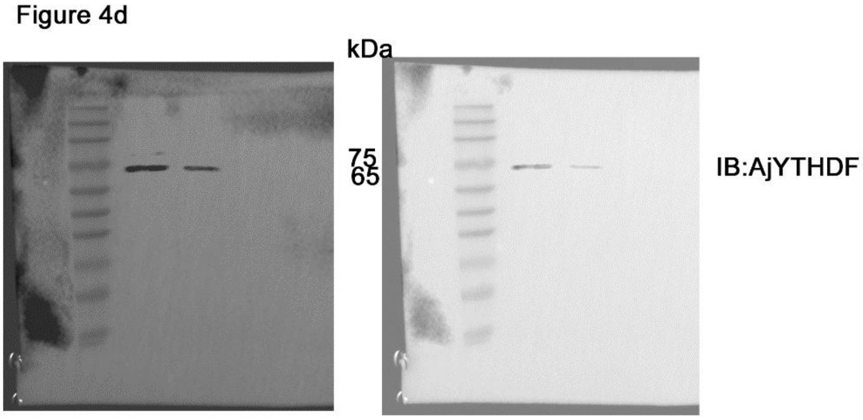

Supplementary Figure 10. Western blots of Figure 4d.

Figure 4f

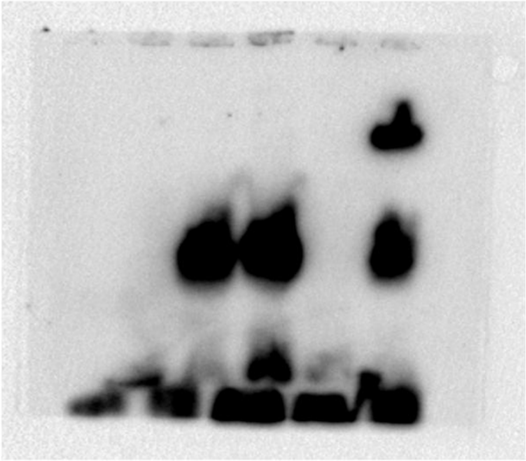

Supplementary Figure 11. RNA blots of Figure 4f.

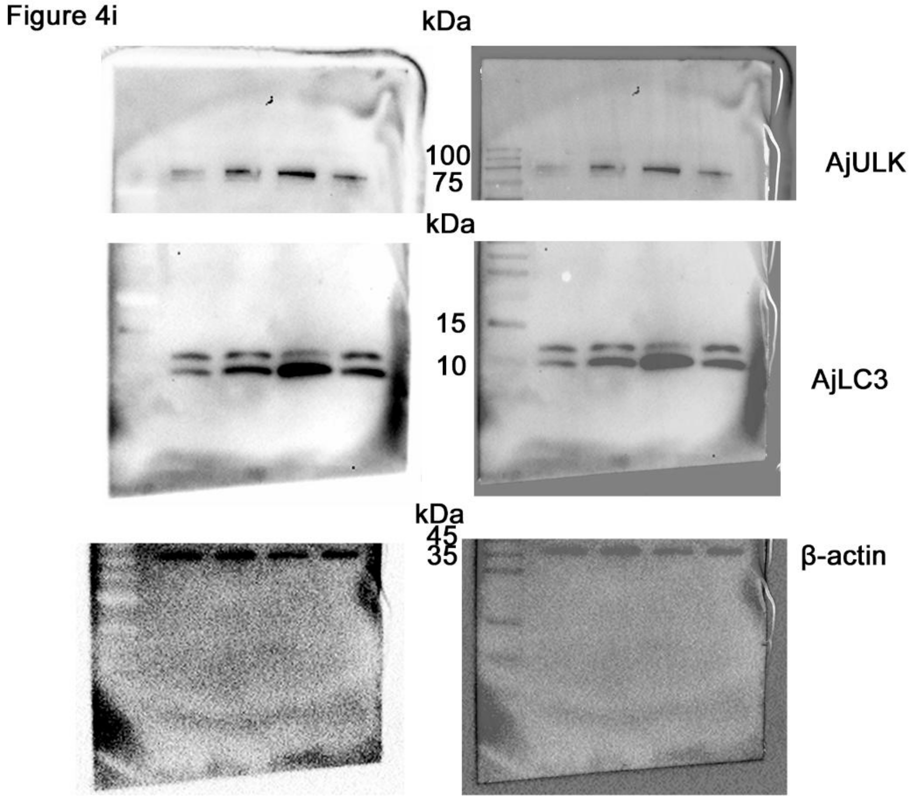

Supplementary Figure 12. Western blots of Figure 4i. The blot image is from same blot membranes.

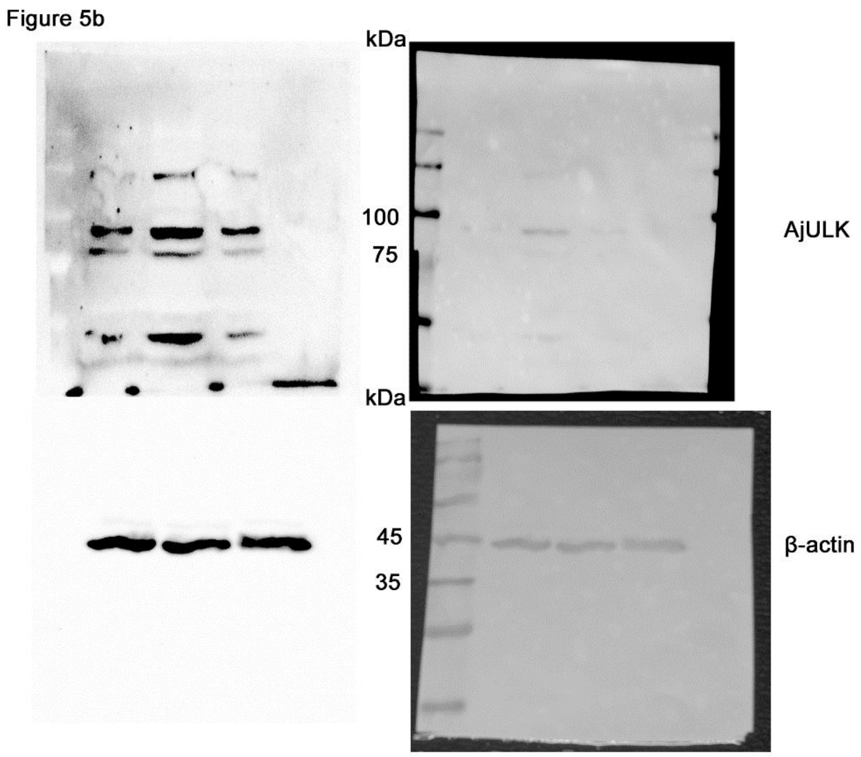

Supplementary Figure 13. Western blots of Figure 5b. The blot image is from two blot membranes.

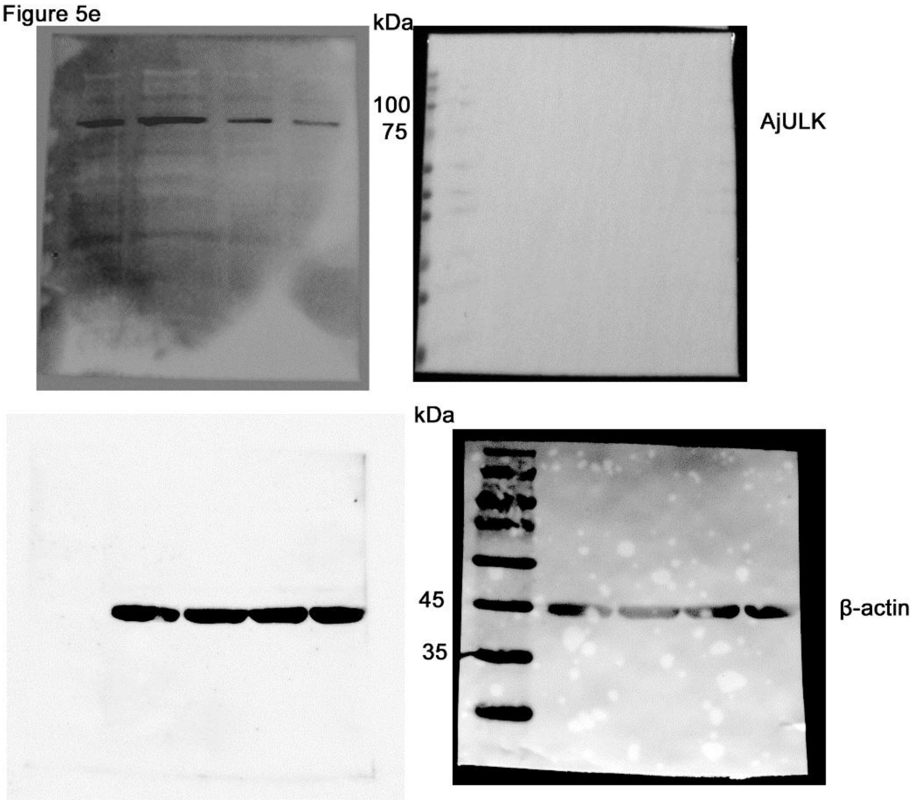

Supplementary Figure 14. Western blots of Figure 5e. The blot image is from two blot membranes.

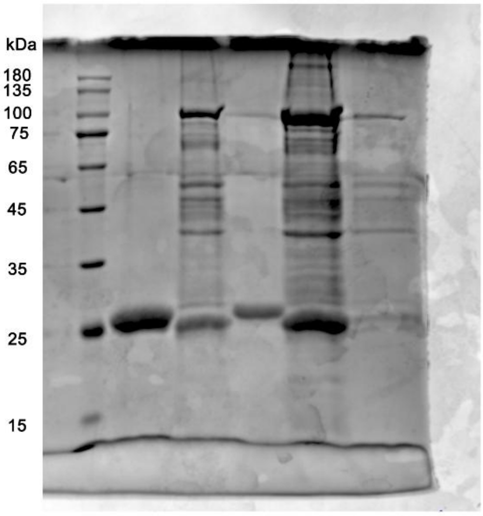

Supplementary Figure 15. GST pull-down of Figure 6a.

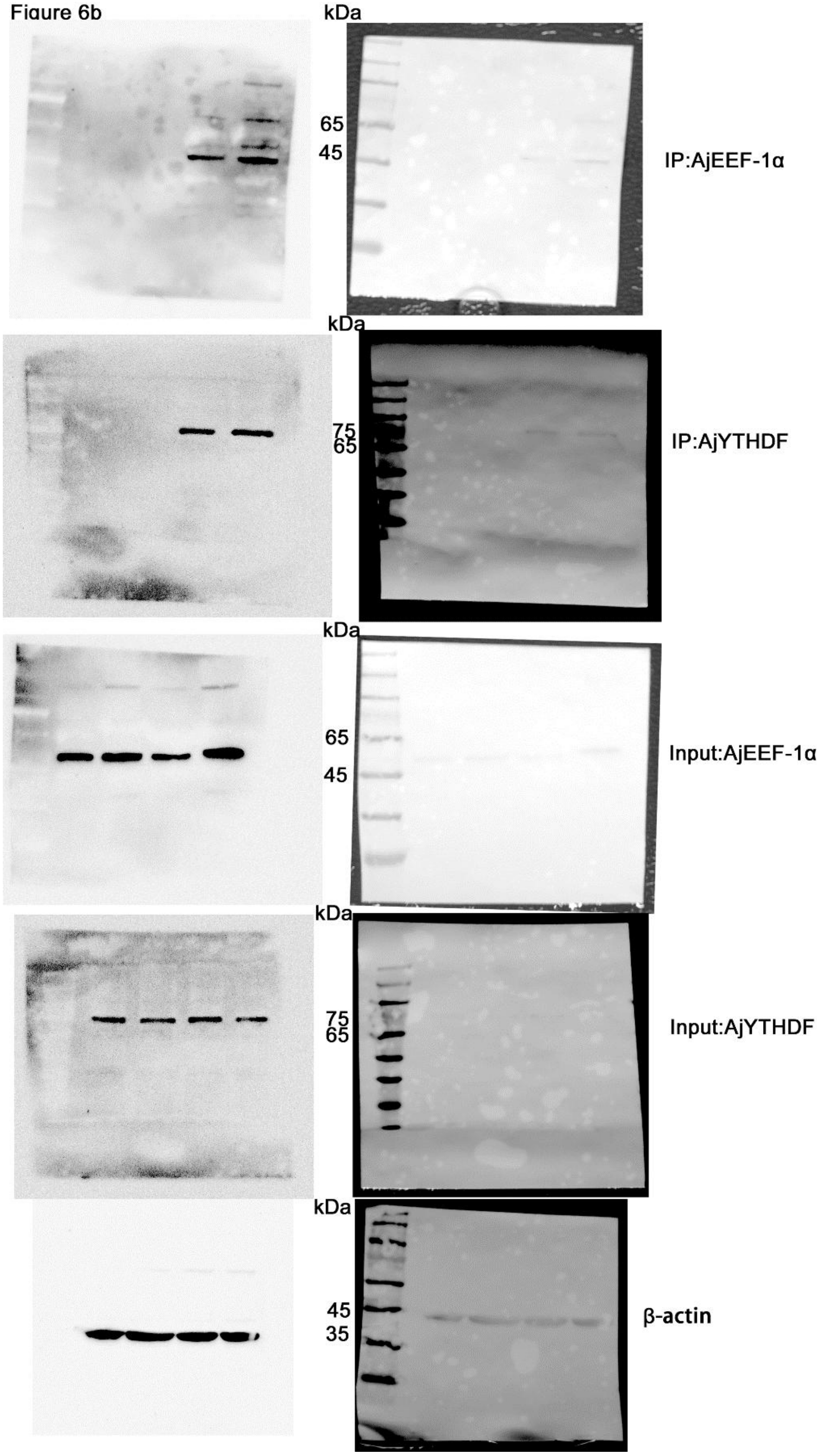

Supplementary Figure 16. Western blots of Figure 6b. The blot image is from five blot membranes.

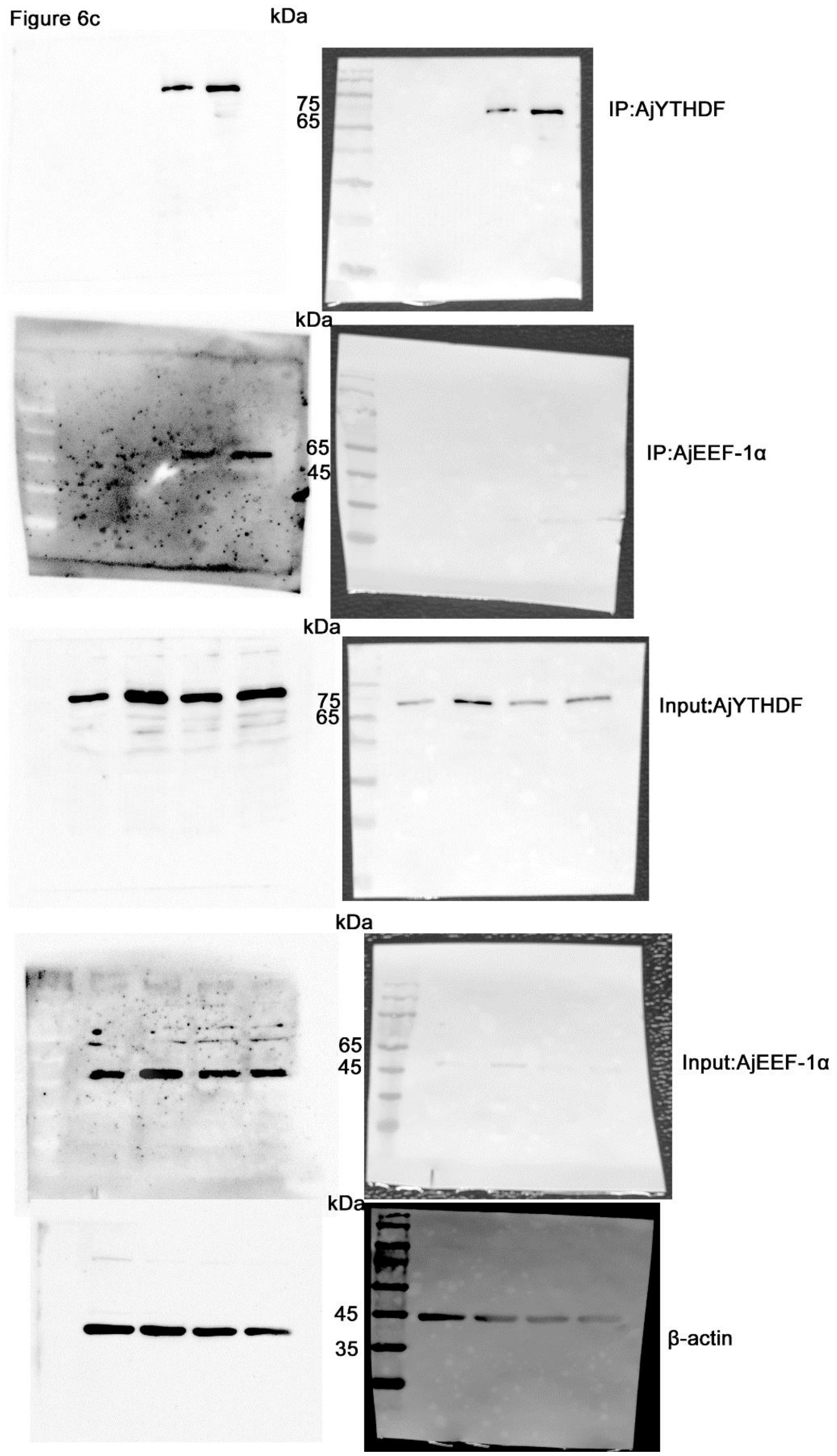

Supplementary Figure 17. Western blots of Figure 6c. The blot image is from five blot membranes.

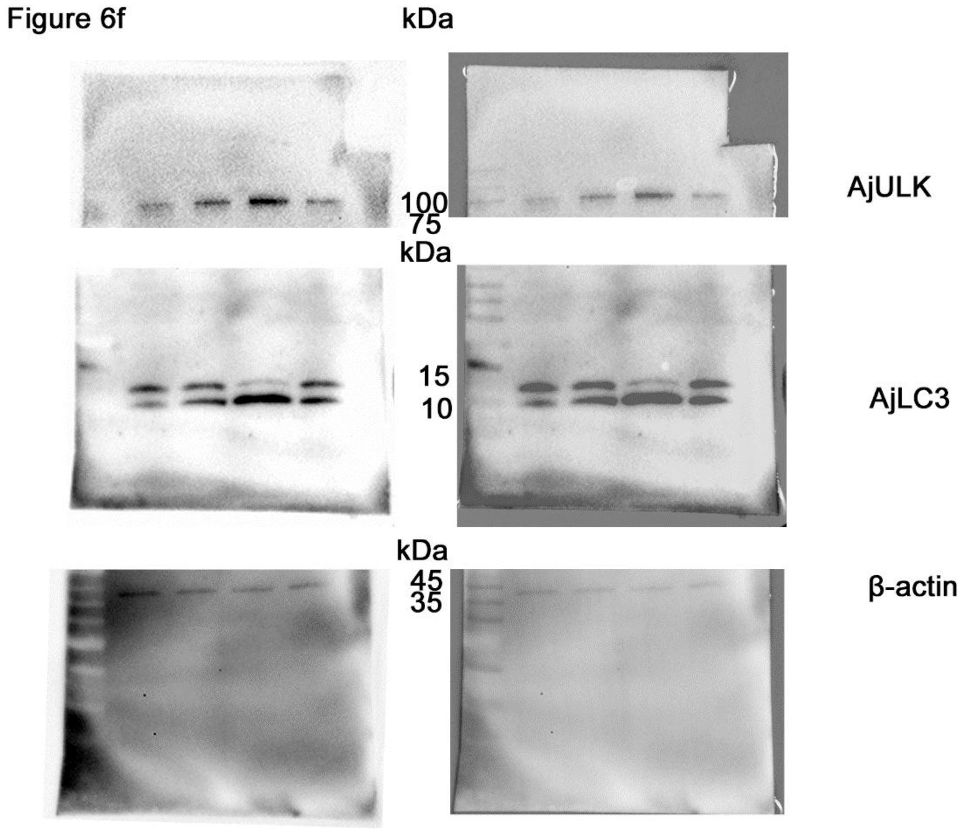

Supplementary Figure 18. Western blots of Figure 6f. The blot image is from same blot membranes.

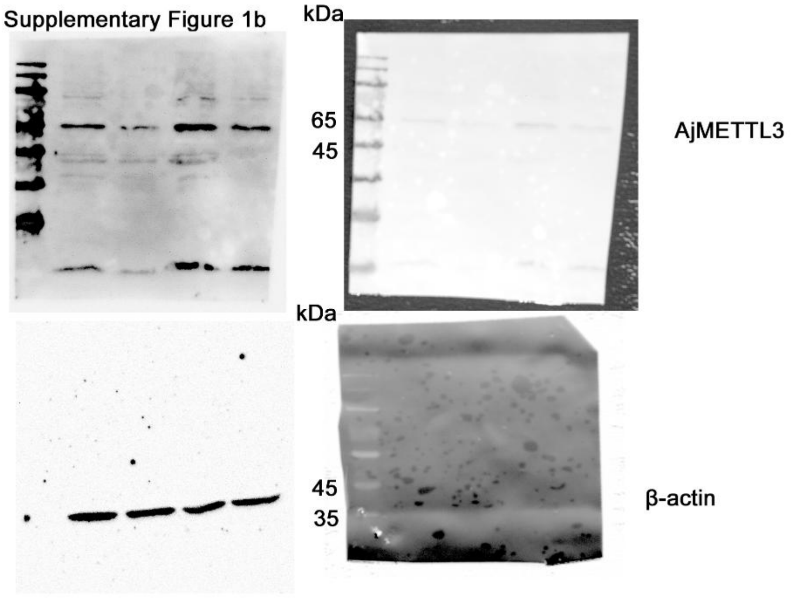

Supplementary Figure 19. Western blots of Supplementary Figure 1b. The blot image is from two blot membranes.
