# Supplementary material for: N6-methyladenosine helps Apostichopus japonicus resist Vibrio splendidus infection by targeting coelomocyte autophagy via the AjULK-AjYTHDF/AjEEF-1α axis
Source: Commun Biol. 2023 May 20;6:547. doi: 10.1038/s42003-023-04929-5 (PMC10199899; doi:10.1038/s42003-023-04929-5)
Supplement: Supplementary file 5 — Reporting Summary [file 42003_2023_4929_MOESM5_ESM.pdf]

nature portfolio

Corresponding author(s): Chenghua Li

Last updated by author(s): May 7, 2023

Reporting Summary

Nature Portfolio wishes to improve the reproducibility of the work that we publish. This form provides structure for consistency and transparency in reporting. For further information on Nature Portfolio policies, see our Editorial Policies and the Editorial Policy Checklist.

Statistics

For all statistical analyses, confirm that the following items are present in the figure legend, table legend, main text, or Methods section.

n/a	Confirmed	
☐	☒	The exact sample size (*n*) for each experimental group/condition, given as a discrete number and unit of measurement
☐	☒	A statement on whether measurements were taken from distinct samples or whether the same sample was measured repeatedly
☐	☒	The statistical test(s) used AND whether they are one- or two-sided<br>*Only common tests should be described solely by name; describe more complex techniques in the Methods section.*
☒	☐	A description of all covariates tested
☐	☒	A description of any assumptions or corrections, such as tests of normality and adjustment for multiple comparisons
☐	☒	A full description of the statistical parameters including central tendency (e.g. means) or other basic estimates (e.g. regression coefficient) AND variation (e.g. standard deviation) or associated estimates of uncertainty (e.g. confidence intervals)
☐	☒	For null hypothesis testing, the test statistic (e.g. *F*, *t*, *r*) with confidence intervals, effect sizes, degrees of freedom and *P* value noted<br>*Give P values as exact values whenever suitable.*
☒	☐	For Bayesian analysis, information on the choice of priors and Markov chain Monte Carlo settings
☐	☒	For hierarchical and complex designs, identification of the appropriate level for tests and full reporting of outcomes
☐	☒	Estimates of effect sizes (e.g. Cohen's *d*, Pearson's *r*), indicating how they were calculated

Our web collection on statistics for biologists contains articles on many of the points above.

Software and code

Policy information about availability of computer code

Data collection	No commercial, open source and custom code were involved in this study.
Data analysis	No commercial, open source and custom code were involved in this study.

For manuscripts utilizing custom algorithms or software that are central to the research but not yet described in published literature, software must be made available to editors and reviewers. We strongly encourage code deposition in a community repository (e.g. GitHub). See the Nature Portfolio guidelines for submitting code & software for further information.

Data

Policy information about availability of data

All manuscripts must include a data availability statement. This statement should provide the following information, where applicable:

- Accession codes, unique identifiers, or web links for publicly available datasets
- A description of any restrictions on data availability
- For clinical datasets or third party data, please ensure that the statement adheres to our policy

The data that support the findings of this study are available in the methods and/or supplementary material of this article. NCBI Accession: AjMETTL3: OQ863733; AjULK: MH807452; AjYTHDF: OQ401099, AjEEF-1α: OQ401098. The m6A-seq results had been uploaded to online repositories with accession number of PRJNA774950 (https://www.ncbi.nlm.nih.gov/pmc/?term=PRJNA774950%20). The Figure 7 was constructed by FigDraw (www.figdraw.com/, ID: OTARUffa00).

Uncropped Western blot images are provided in Supplementary Figure 5-19. All statistical source data that underlie the graphs in figures are provided in Supplementary Data 1.

Human research participants

Policy information about studies involving human research participants and Sex and Gender in Research.

Reporting on sex and gender	No human research was involved in this study.
Population characteristics	No human research was involved in this study.
Recruitment	No human research was involved in this study.
Ethics oversight	No human research was involved in this study.

Note that full information on the approval of the study protocol must also be provided in the manuscript.

Field-specific reporting

Please select the one below that is the best fit for your research. If you are not sure, read the appropriate sections before making your selection.

☒ Life sciences ☐ Behavioural & social sciences ☐ Ecological, evolutionary & environmental sciences

For a reference copy of the document with all sections, see nature.com/documents/nr-reporting-summary-flat.pdf

Life sciences study design

All studies must disclose on these points even when the disclosure is negative.

Sample size	Healthy adult sea cucumber A. japonicus (weight: 98 ± 11 g).
Data exclusions	No data were exclude from the analyses in this study.
Replication	Each experiment was performed for three times, and all attempts at replication were successfullly.
Randomization	The experimental animals were randomly collected from each group in this study.
Blinding	The investigators were blinded to group allocation during data collection or analysis in this study.

Reporting for specific materials, systems and methods

We require information from authors about some types of materials, experimental systems and methods used in many studies. Here, indicate whether each material, system or method listed is relevant to your study. If you are not sure if a list item applies to your research, read the appropriate section before selecting a response.

Materials & experimental systems

n/a	Involved in the study	
☐	☒	Antibodies
☐	☒	Eukaryotic cell lines
☒	☐	Palaeontology and archaeology
☐	☒	Animals and other organisms
☒	☐	Clinical data
☒	☐	Dual use research of concern

Methods

n/a	Involved in the study	
☒	☐	ChIP-seq
☒	☐	Flow cytometry
☒	☐	MRI-based neuroimaging

Antibodies

Antibodies used	Mouse against A. japonicus METTL3,YTHDF,ATG13,Beclin-1 polyclonal antibody was performed by ourselves. The commercial antibodies:LAMP primary monoclonal antibody （Beyotime AG2482, Shanghai, China) , AjULK antibody （Abmart T56902S， Shanghai， China), Ajp62 antibody (Klean AB P113161, Shanghai, China), AjLC3 antibody (Abcam EPR18709,Cambridge,Britain), AjEEF-1α antibody (Betotime AF6765, Shanghai, China), β-actin polyclonal antibody (KleanAB P100003, Shanghai, China).

Validation	For the validation of METTL3,YTHDF,ATG14,Beclin-1 antibody specificity, we performed the recombinant and purified these proteins. The soluble target proteins were injected into 6-week-old mice to acquire polyclonal antibodies according to our previously protocol. In addition, the specificity of commercial antibodies were verified by total protein of sea cucumber coelomocytes.

Eukaryotic cell lines

Policy information about cell lines and Sex and Gender in Research

Cell line source(s)	the epithelioma papulosum cyprinid (EPC)
Authentication	The EPC line was purchased from China Zebrafish Resource Center (CZRC).
Mycoplasma contamination	We confirmed that all cell lines tested negative for mycoplasma contamination.
Commonly misidentified lines (See ICLAC register)	We did not name any commonly misidentified cell lines used in the study.

Animals and other research organisms

Policy information about studies involving animals; ARRIVE guidelines recommended for reporting animal research, and Sex and Gender in Research

Laboratory animals	Sea cucumber, A. japonicus, two years old.
Wild animals	No wild animals were involved in this study.
Reporting on sex	The sex of the experimental animals was not considered in this study.
Field-collected samples	The study did not involve samples collected from the field.
Ethics oversight	The sea cucumbers A. japonicus used in this work were commercially cultured animals, and all experiments were conducted in accordance with the recommendations in the Guide for the Care and Use of Laboratory Animals of the National Institutes of Health. The study protocol was approved by the Experimental Animal Ethics Committee of Ningbo University, China (No. NBU-ES-2021-11180).

Note that full information on the approval of the study protocol must also be provided in the manuscript.
